# An international multi-institutional validation of T1 sub-staging of intraductal papillary mucinous neoplasm-derived pancreatic cancer

**DOI:** 10.1093/jnci/djae166

**Published:** 2024-07-19

**Authors:** Joseph R Habib, Ingmar F Rompen, Brady A Campbell, Paul C M Andel, Benedict Kinny-Köster, Ryte Damaseviciute, D Brock Hewitt, Greg D Sacks, Ammar A Javed, Marc G Besselink, Hjalmar C van Santvoort, Lois A Daamen, Martin Loos, Jin He, I Quintus Molenaar, Markus W Büchler, Christopher L Wolfgang

**Affiliations:** Department of Surgery, New York University Langone Health, New York, NY, USA; Department of Surgery, University Medical Center Utrecht, Utrecht, The Netherlands; Department of Surgery, Regional Academic Cancer Center Utrecht, Utrecht, The Netherlands; Department of Surgery, New York University Langone Health, New York, NY, USA; Department of General, Visceral and Transplantation Surgery, Heidelberg University Hospital, Heidelberg, Germany; Department of Surgery, Johns Hopkins Hospital, Baltimore, MD, USA; Department of Surgery, University Medical Center Utrecht, Utrecht, The Netherlands; Department of Surgery, Regional Academic Cancer Center Utrecht, Utrecht, The Netherlands; Department of General, Visceral and Transplantation Surgery, Heidelberg University Hospital, Heidelberg, Germany; Department of Surgery, New York University Langone Health, New York, NY, USA; Department of Surgery, New York University Langone Health, New York, NY, USA; Department of Surgery, New York University Langone Health, New York, NY, USA; Department of Surgery, New York University Langone Health, New York, NY, USA; Department of Surgery, Amsterdam UMC, location University of Amsterdam, Amsterdam, The Netherlands; Cancer Center Amsterdam, Amsterdam, The Netherlands; Department of Surgery, University Medical Center Utrecht, Utrecht, The Netherlands; Department of Surgery, Regional Academic Cancer Center Utrecht, Utrecht, The Netherlands; Department of Surgery, University Medical Center Utrecht, Utrecht, The Netherlands; Division of Imaging and Oncology, University Medical Center Utrecht, Utrecht, The Netherlands; Department of General, Visceral and Transplantation Surgery, Heidelberg University Hospital, Heidelberg, Germany; Department of Surgery, Johns Hopkins Hospital, Baltimore, MD, USA; Department of Surgery, University Medical Center Utrecht, Utrecht, The Netherlands; Department of Surgery, Regional Academic Cancer Center Utrecht, Utrecht, The Netherlands; Department of Surgery, Champalimaud Foundation, Lisbon, Portugal; Department of Surgery, New York University Langone Health, New York, NY, USA

## Abstract

**Background:**

Intraductal papillary mucinous neoplasm (IPMN)–derived pancreatic ductal adenocarcinoma (PDAC) is resected at smaller sizes compared with its biologically distinct counterpart, pancreatic intraepithelial neoplasia (PanIN)–derived PDAC. Thus, experts proposed T1 sub-staging for IPMN-derived PDAC. However, this has never been validated.

**Methods:**

Consecutive upfront surgery patients with IPMN-derived PDAC from 5 international high-volume centers were classified by the proposed T1 sub-staging classification (T1a ≤0.5, T1b >0.5 and ≤1.0, and T1c >1.0 and ≤2.0 cm) using the invasive component size. Kaplan-Meier and log-rank tests were used to compare overall survival (OS). A multivariable Cox regression was used to determine hazard ratios (HRs) with confidence intervals (95% CIs).

**Results:**

Among 747 patients, 69 (9.2%), 50 (6.7%), 99 (13.0%), and 531 patients (71.1%), comprised the T1a, T1b, T1c, and T2-4 subgroups, respectively. Increasing T-stage was associated with elevated CA19-9, poorer grade, nodal positivity, R1 margin, and tubular subtype. Median OS for T1a, T1b, T1c, and T2-4 were 159.0 (95% CI = 126.0 to NR), 128.8 (98.3 to NR), 77.6 (48.3 to 108.2), and 31.4 (27.5 to 37.7) months, respectively (*P* < .001). OS decreased with increasing T-stage for all pairwise comparisons (all *P* < .05). After risk adjustment, older than age 65, elevated CA19-9, T1b [HR = 2.55 (1.22 to 5.32)], T1c [HR = 3.04 (1.60 to 5.76)], and T2-4 [HR = 3.41 (1.89 to 6.17)] compared with T1a, nodal positivity, R1 margin, and no adjuvant chemotherapy were associated with worse OS. Disease recurrence was more common in T2-4 tumors (56.4%) compared with T1a (18.2%), T1b (23.9%), and T1c (36.1%, *P* < .001).

**Conclusion:**

T1 sub-staging of IPMN-derived PDAC is valid and has significant prognostic value. Advancing T1 sub-stage is associated with worse histopathology, survival, and recurrence. T1 sub-staging is recommended for future guidelines.

Pancreatic ductal adenocarcinoma (PDAC) most commonly arises from pancreatic intraepithelial neoplasia (PanIN) followed by intraductal papillary mucinous neoplasm (IPMN) precursor lesions (1). Because of a lack of evidence-based studies for IPMN-derived PDAC, management of these tumors is often based on data extrapolated from studies of PanIN-derived PDAC. However, mounting evidence suggests 2 biologically distinct entities with dissimilar oncological outcomes (2-5).

IPMN-derived PDAC generally has more favorable outcomes compared with PanIN-derived PDAC. Possible reasons include earlier interventions secondary to preoperative surveillance of a known cyst, more indolent disease biology, or an earlier onset of symptoms because of a mass effect as IPMN-derived PDAC is often accompanied by a large noninvasive cystic component (6). In some series, it is estimated that almost 50% of IPMN-derived PDAC are resected with a tumor size that is less than 2 centimeters (cm), corresponding to a T1 staged cancer (7-9). Alternatively, in PanIN-derived PDAC, only 7%-18% are resected at T1 stage (10-12). Nevertheless, both IPMN and PanIN-derived PDAC are currently staged using the same American Joint Committee on Cancer (AJCC) classification to determine prognosis and inform treatment decisions (13).

Because of the greater incidence of resected T1 PDACs arising from IPMNs, the international consensus meeting for IPMNs proposed a T1 sub-staging classification (T1a ≤0.5, T1b >0.5 and ≤1.0, and T1c >1.0 and ≤2.0 cm) to improve prognostication (14,15). However, the proposed T1 sub-staging system has not been validated. The central aim of this study was to leverage the large multicenter experiences at multiple international high-volume centers to evaluate the performance and prognostic value of the proposed T1 sub-staging classification system for IPMN-derived PDAC.

## Methods

### Study population

Patients who underwent pancreatic resection and were found to have IPMN-derived PDAC between 2000 and 2021 were identified from institutional databases at 5 international high-volume centers including UMC Amsterdam, Heidelberg University Hospital, Johns Hopkins Hospital, New York University Langone Health, and the Academic Cancer Center Utrecht.

Patients with concomitant PDAC separate from an IPMN, those who underwent neoadjuvant therapy, had missing tumor size, a grossly positive resection margin (R2), oncocytic tumors (16), or 90-day postoperative mortality were excluded. Institutional review board approval was obtained by all participating centers, and this study complied with all Health Insurance Portability and Accountability Act regulations. The strengthening and reporting of observational studies in epidemiology (STROBE) guidelines were used.

### Pathological assessment and variable definitions

Histopathology was reviewed by a local pancreas pathologist at each center to confirm that the invasive carcinoma was arising from an IPMN and not a concomitant PDAC lesion. Specimens were rereviewed by these pathologists to ensure that direct association of the IPMN with the invasive component was present. Patients where no direct association (no clear pathological connection) between the IPMN and invasive component was observed were excluded. However, in accordance with the international consensus guidelines, the combination of intestinal IPMN and colloid carcinoma implied that the invasive component was IPMN-derived because concomitant PDAC exclusive shows tubular carcinoma (15). T-stage was evaluated using the size of the invasive component in accordance with the updated international consensus guidelines (15). This was routinely assessed microscopically in the cases of smaller tumors. T1 tumors were further sub-staged according to the proposed classification: T1a ≤0.5, T1b >0.5 and ≤1.0, and T1c >1.0 and ≤2.0 cm. IPMN-derived cancers were characterized as colloid or tubular histological subtypes. Nodal disease was stratified on the basis of the presence or absence of disease. The resection margin was defined as R1 when there was microscopic evidence of invasive cancer at or within 1 millimeter of the resection margin. Preoperative CA19-9 values were used to stratify patients into 4 cohorts: nonproducers (17) CA19-9 <5 u/mL, normal CA19-9 (>5 to ≤37 u/mL), elevated CA19-9 (>37 u/mL), and unknown. The AJCC TNM system, 8th edition, was used for staging (13). Decisions regarding the use of adjuvant chemotherapy were institutionally dependent and were most often made in a multidisciplinary setting. Initial site of recurrence was used to define the pattern of recurrence. Local recurrence included recurrence of disease in the pancreatic remnant, surgical bed, peripancreatic lymph nodes, or surrounding vasculature, whereas systemic recurrence was defined as recurrence in the liver, lung, peritoneum, or any other distant site (7,18).

### Statistical analysis

Categorical variables were displayed as frequencies and percentages and were compared using a χ^2^ test. The date of surgery was used as time zero for all time-to-event analyses. Overall survival (OS) was defined as time between surgical resection and date of death or censored at last known follow-up for patients found to be alive. Recurrence-free survival (RFS) was defined from the date of surgery to the date of recurrence or death, whichever came first. Survival follow-up was truncated after 10 years because of a limited number of patients at risk after this point. Kaplan-Meier analysis was performed for all time-to-event analyses, and these were used to derive the median OS and RFS for all patients, and then for each T1 sub-stage cohort. Univariable log-rank tests were used to compare overall and paired Kaplan-Meier curves. A subanalysis using Kaplan-Meier plots and log-rank tests was performed on ductal type and within T1 sub-stage cohorts. RFS and patterns of recurrence were compared using Kaplan-Meier curves and log-rank tests and by χ^2^ test, respectively. A backward selection Cox regression employing T1 sub-stages was used for multivariable risk adjustment with corresponding risk-adjusted hazard ratios (HRs) and 95% confidence intervals (CIs) to validate the prognostic ability T1 sub-stage. An additional multivariable Cox regression was performed to further assess prognostic impact of tumor size as continuous variable (per 1 cm). A 2-sided *P* value less than .05 was used to determine statistical significance. Statistical analysis was performed with the “R” statistical software (version 4.2.3) using the “survminer,” “survival,” and “ggplot2” packages.

## Results

### Study population and clinicopathological data

The study population consisted of 747 patients with IPMN-derived PDAC. Of these, 69 (9.2%), 50 (6.7%), 97 (13.0%), and 531 patients (71.1%) made up the T1a, T1b, T1c, and T2-4 subgroups, respectively. Of the 216 (28.9%) T1 cancers, 31.9% were T1a, 23.1% were T1b, and the remaining 44.9% were T1c. Table 1 summarizes the demographic and clinicopathological characteristics of each cohort. On univariable analysis, increasing T-stage was significantly associated with elevated CA19-9 (*P* < .001), poorer grade of differentiation (0.007), perineural invasion (*P* < .001), nodal positivity (<.001), R1 margin (*P* < .001), and tubular subtype (*P* < .001). Age (*P* = .772), sex (*P* = .882), ductal type (*P* = .214), and type of surgical operation (*P* = .838) were similar between the 4 cohorts (Table 1).

**Table 1.** Basic demographics and clinicopathological information for all patients stratified by T-stage with corresponding *P* values on the basis of univariable comparisons

Variable	T1a (n = 69) n (%)	T1b (n = 50) n (%)	T1c (n = 97) n (%)	T2-4 (n = 531) n (%)	*P*
Age >65	44 (64)	32 (64)	67 (69)	365 (69)	.772
Female	32 (46)	24 (48)	50 (52)	251 (47)	.882
Preoperative CA19-9					
Elevated	12 (27)	18 (44)	47 (53)	288 (66)	<.001
Normal	32 (71)	22 (54)	36 (41)	125 (29)	
Nonproducer	1 (2)	1 (2)	5 (5)	22 (5)	
Unknown	24	9	9	96	
Operation					
Pancreatoduodenectomy	36 (52)	30 (60)	51 (53)	291 (55)	.838
Distal pancreatectomy	17 (25)	10 (20)	20 (21)	98 (18)	
Total pancreatectomy	16 (23)	10 (20)	26 (27)	142 (27)	
Duct type					.214
Main	24 (38)	13 (29)	28 (32)	125 (29)	
Branch	13 (20)	14 (31)	12 (14)	83 (19)	
Mixed type	27 (42)	18 (40)	47 (54)	224 (52)	
Unknown	5	5	10	99	
R1 margin	1 (1)	2 (4)	20 (21)	257 (48)	<.001
Poor grade of differentiation	11 (16)	7 (14)	21 (22)	151 (30)	.007
Tubular subtype	39 (62)	28 (62)	70 (76)	395 (81)	<.001
Perineural invasion	6 (11)	9 (31)	23 (47)	166 (72)	<.001
Positive nodes	2 (3)	8 (16)	42 (43)	348 (66)	<.001
Adjuvant chemotherapy	22 (38)	21 (53)	50 (65)	269 (71)	<.001

Individual univariable comparisons between T1 sub-staged groups are summarized in Supplementary Tables 1-3 (available online). Briefly, compared with T1a, T1b cancers were associated with elevated CA19-9 (*P* = .047), presence of nodal disease (*P* = .016), and perineural invasion (*P* = .027), whereas T1c cancers were associated with elevated CA19-9 (*P* = .006), nodal disease (*P* < .001), R1 margins (*P* < .001), and perineural invasion (*P* < .001). T1c patients were more likely to receive adjuvant chemotherapy (*P* = .002) than T1a patients. Compared with T1b, T1c cancers were associated with worse nodal disease (*P* = .001) and R1 margins (*P* = .007).

### Overall survival by T-stage

**Figure 1.**
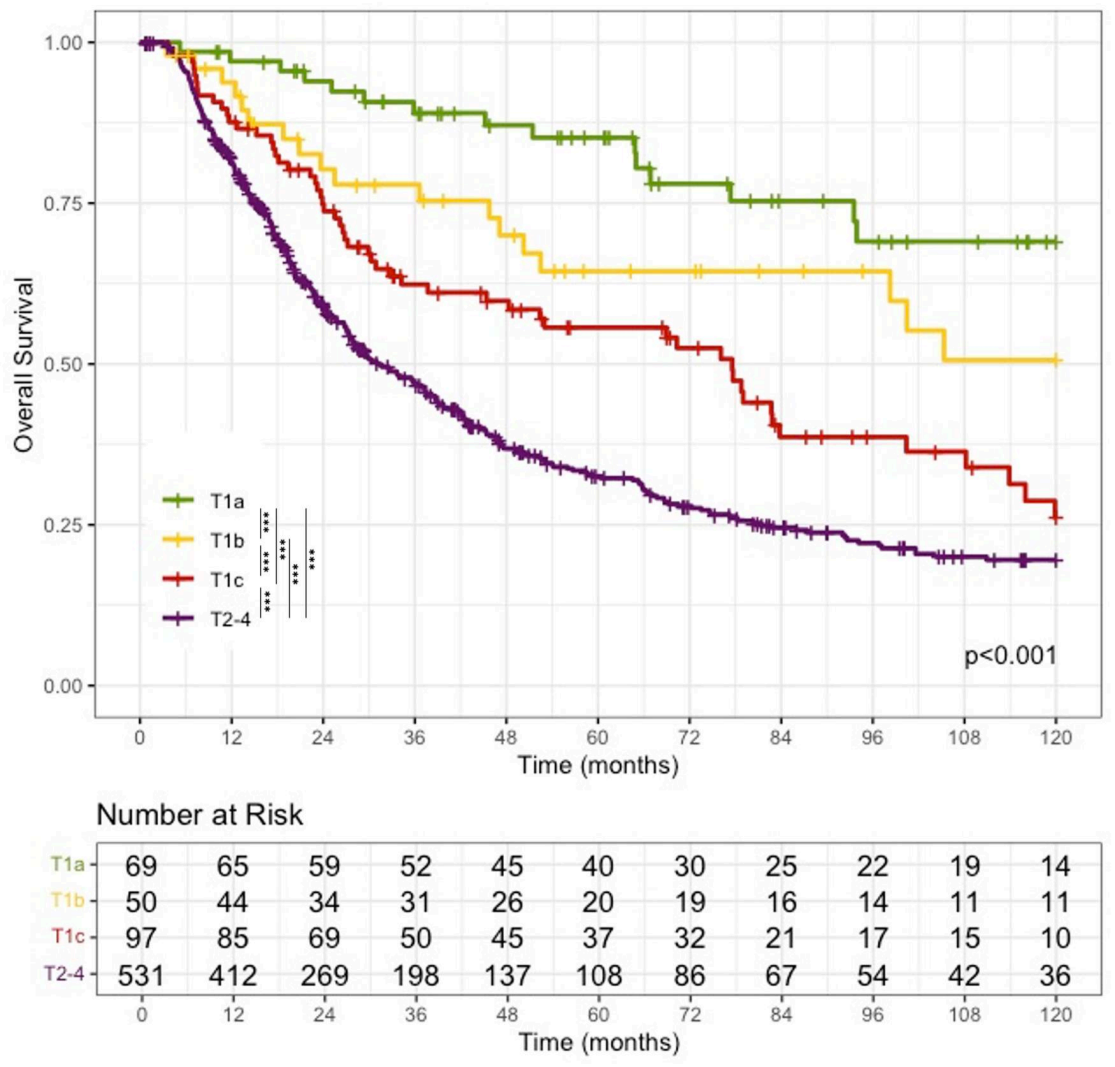
Overall survival by T-stage. Kaplan-Meier survival curves and log-rank test for overall survival stratified by T-stage. All pairwise log-rank *P* values less than .05 (***).

The median OSs for T1a, T1b, T1c, and T2-4 were 159.0 (95% CI = 126.0 to NR), 128.8 (98.3 to NR), 77.6 (48.3 to 108.2), and 31.4 (27.5 to 37.7) months, respectively (*P* < .001). Kaplan-Meier survival curves are illustrated in Figure 1. On pairwise unadjusted log-rank comparisons, patients with T1a cancers had significantly longer survival than those with T1b (*P* = .049), T1c (*P* < .001), and T2-4 (*P* < .001). T1b patients had longer survival than T1c (*P* = .045) and T2-4 (*P* < .001) cancers. T1c patients had longer survival than T2-4 cancers (*P* < .001). Overall survival was similar across all institutions (*P* = .376). Within T1 sub-stages and T2-4 tumors, OS did not differ significantly based on ductal type (branch duct vs main duct vs mixed type) (Supplementary Figure 1, available online, *P* > .05 within each T-stage class).

### Disease recurrence by T-stage

Of the 603 patients with complete recurrence data, 283 (46.9%) developed disease recurrence with a median follow-up of 49.0 months (interquartile range [IQR] = 21.6-89.3) in surviving patients. The median RFS of T1a, T1b, T1c, and T2-4 tumors was not reached (93.3 to not reached), 104.9 (45.6 to not reached), 54.8 (29.8 to 79.7), and 18.0 (15.8 to 20.2) months, respectively (*P* < .001). Of those that recurred, 196 (69.3%) had a systemic recurrence, whereas the remaining 87 (30.7%) had local-only first recurrence. Of T1a, T1b, T1c, and T2-4 tumors, 18.2%, 23.9%, 36.1%, and 56.4% experienced disease recurrence, respectively (*P* < .001). Particularly, systemic recurrence was increasingly more common in T2-4 tumors (39.7%) than in T1a (12.1%), T1b (17.4%), and T1c (21.7%) tumors (*P* < .001). Similarly, the incidence of local-only recurrence was increasingly more common among more advanced T-stages (T1a: 6.1%, T1b: 6.5%, T1c: 14.5%, and T2-4: 16.7%, *P* = .052). In patients with an R0 resection, the rate of local recurrence in T1a, T1b, T1c, and T2-4 tumors was 6.2%, 6.7%, 17.4%, and 14.2%, respectively (*P* = .118).

### Multivariable analysis for overall survival

**Figure 2.**
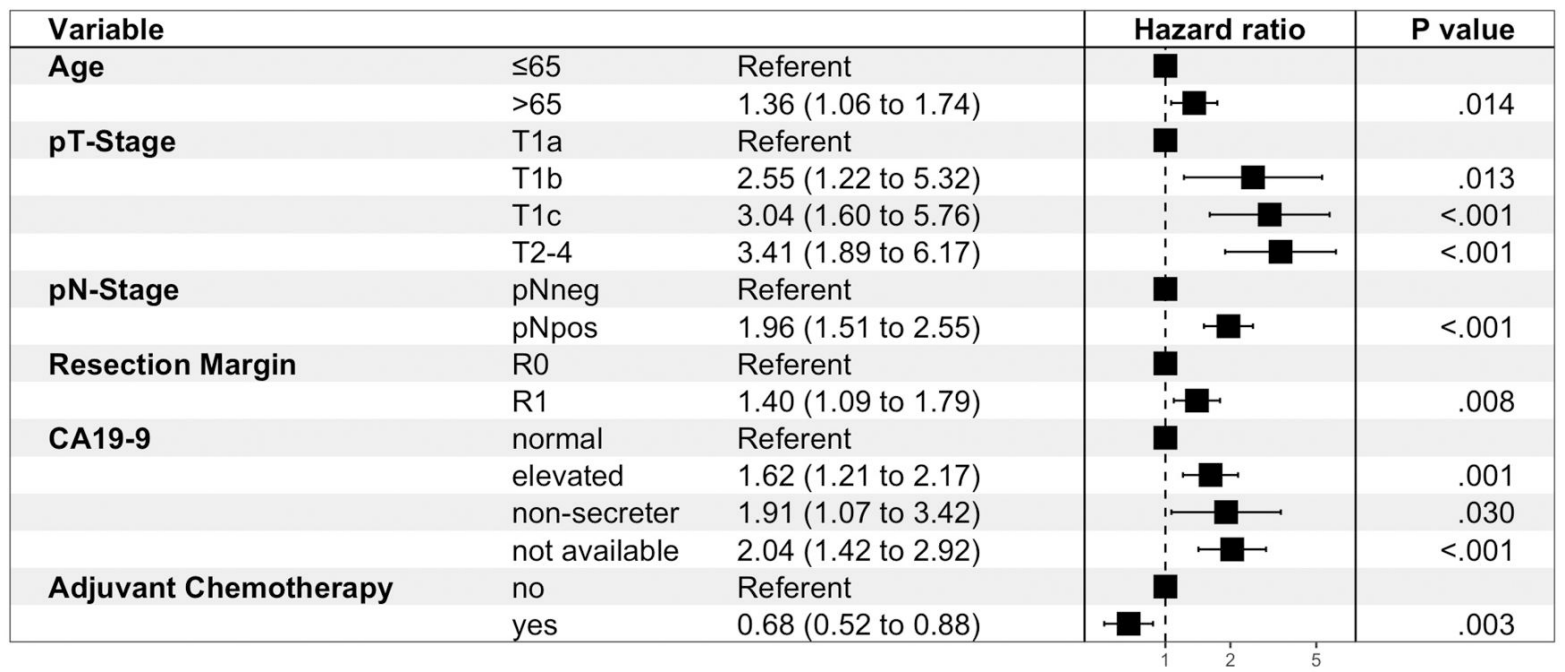
Multivariable Cox regression for overall survival. Forest plot depicting hazard ratios and 95% confidence intervals of variables statistically significant on Cox regression analysis.

On multivariable Cox regression analysis, age older than 65 years [HR = 1.36 (1.06 to 1.74)], elevated CA19-9 [HR = 1.62 (1.21 to 2.17)], T1b [HR = 2.55 (1.22 to 5.32)], T1c [HR = 3.04 (1.60 to 5.76)], and T2-4 [HR = 3.41 (1.89 to 6.17)] compared with T1a, nodal positivity [HR = 1.96 (1.51 to 2.55)], and R1 margin [HR = 1.40 (1.09 to 1.79)] were associated with worse OS. Alternatively, adjuvant chemotherapy [HR = 0.68 (0.52 to 0.88)] was protective and was associated with improved OS. Figure 2 displays a forest plot summarizing these results. On multivariable Cox regression for all patients, a 1 cm increase in tumor size [HR = 1.06 (1.03 to 1.10)] was significantly associated with worse OS (Supplementary Figure 2, available online).

### Adjuvant chemotherapy based on T-stage

Adjuvant chemotherapy was administered more often with increasing T sub-stage (T1a: 38%, T1b: 53%, T1c: 65%, T2-4: 71%, *P* < .001) with a similar number of completed cycles (median = 6, *P* = .655). Adjuvant chemotherapy administration practices differed significantly between institutions (range: 76%-41%, *P* < .001). Gemcitabine-based chemotherapy regimens were more often administered than 5FU-based chemotherapy (90% vs 10%, *P* = .001). No associated benefit in OS was observed after administering adjuvant chemotherapy in T1a (*P* = .178), T1b (*P* = .314), or T1c (*P* = .130) tumors. However, patients with T2-4 tumors who received adjuvant chemotherapy (mOS: 39.5 months, 95% CI = 32.8 to 47.6) had a significantly longer OS compared with those who did not (mOS: 22.7 months, 95% CI = 16.9 to 29.8, *P* = .010; Supplementary Figure 3, available online).

## Discussion

Currently, the AJCC 8th edition staging system, predominantly derived from data on PanIN-derived PDAC, is also applied to patients with IPMN-derived PDAC. Recent literature suggests that these entities are biologically divergent (2-5). Furthermore, IPMN-derived PDAC is resected at considerably smaller tumor sizes than its PanIN counterpart. Thus, a distinct staging system is needed to improve prognostication in this large proportion of resected T1 IPMN-derived PDAC. Appropriately, content experts have proposed a T1 sub-staging system (T1a ≤0.5, T1b >0.5 and ≤1.0, and T1c >1.0 and ≤2.0 cm) to address this concern. Our study serves as the largest evaluation of this proposed T1 sub-staging system. In this validation, we observed a progressive worsening of histopathological features and disease recurrence with advancing T sub-stage. Furthermore, T sub-stage independently predicted OS.

When considering studies reporting the distribution of T1 sub-stages across all PDAC, the presence of a stage migration becomes evident. A recent study by Kwon et al. (19) evaluating all T1 PDAC in the Surveillance, Epidemiology, and End Results (SEER) program database demonstrated a significant skew toward T1c tumors with 2.3%, 4.1%, and 93.6% of tumors being staged as T1a, T1b, and T1c, respectively. Of note, the study population was a representative sample of the PDAC population—the majority of tumors were PanIN-derived PDAC. Contrastingly, when evaluating a study population comprising patients with IPMN-derived PDAC, T stage distributions are more well balanced, as seen in the current study. Notably, 29.1% of the patients in our study were staged as T1. This frequency is considerably higher compared with patients with PanIN-derived PDAC (7%-18%) (10-12). Moreover, the proportion of T1 IPMN-derived PDACs is even higher than in patients who underwent neoadjuvant therapy for PanIN-derived PDAC and were subsequently selected for surgery after potential downstaging of tumor size (20). Thus, when separating IPMN-derived PDAC from PanIN-derived PDAC, a redistribution of T-stage will likely be observed.

Content experts proposed a T1 sub-staging system specific to IPMN-derived PDAC to improve prognostication in these cancers. In this study, advancing T1 sub-stage of IPMN-derived PDACs was increasingly associated with worse histopathological features in a stepwise manner. Worse features included elevated CA19-9, poorer grade of differentiation, perineural invasion, nodal positivity, and more tubular histology. These data suggest that progressive T1 sub-stage depicts worse tumor biological features. Multivariable analysis showed that T1 sub-staging remained a statistically significant predictor in the final model, indicating an independent contribution to predicting survival. However, Shah et al. (21), who evaluated PanIN-derived PDAC, did not report an association between T-stage subclassification and OS after risk adjustment, again suggesting the need for distinct guidelines and staging systems.

For PanIN-derived PDAC, studies investigating the utility of this subclassification have concluded that a single cutoff of 1 cm to split T1 tumors into 2 groups may have more prognostic value because of the low incidence of tumors less than 1 cm (21,22). Alternatively, we found that the 3-tier staging system has prognostic value with significant pairwise comparisons in overall survival, and this trend remained significant after further adjusting for additional factors associated with survival. Margonis et al. (23) also evaluated T1 sub-staging for IPMN-derived PDAC and recommended combining T1b and T1c tumors for improved prognostication. However, in their study, there was a notable decreasing trend in median OS with worsening T1 sub-stage (T1a: 126 months, T1b: 87.4 months, and T1c: 50.2 months). The significant difference observed between all sub-stages in our study is likely due to the larger population size, and the aforementioned study might have been underpowered to detect a significant difference in survival.

Beyond prognostication, a progressively worsening RFS was observed in patients with more advanced T1 sub-stage. This may indicate that T sub-staging may have a role in developing patient specific surveillance strategies for patients with resected IPMN-derived PDAC. Albeit less frequent, even in T1a tumors, 18% of patients still experienced disease recurrence. Future studies should investigate T1 sub-staging alongside other factors in predicting the need for and optimal intervals for surveillance.

The role of adjuvant chemotherapy for IPMN-derived PDAC is controversial (8, 24-28). This is further highlighted in this study, where the use of adjuvant chemotherapy significantly differed by institution, likely mimicking real-world practice. Herein, we found that adjuvant chemotherapy was associated with an improvement in OS for patients with T2-4 tumors, whereas no associated benefit was observed in any T1 tumor sub-stage. Furthermore, we observed that systemic disease recurrence, as well as clinicopathological factors associated with systemic disease such as elevated CA19-9 and nodal positivity, were increasingly more common among patients with larger T-stages. The observed associated benefit of adjuvant chemotherapy in larger tumor sizes is likely secondary to its necessity in cancers with more aggressive disease biology (increased association with markers of systemic disease such as elevated CA19-9 and nodal disease) that subsequently lead to a propensity for systemic progression (8,24,29).

Several limitations should be acknowledged in this study. First, the inherent bias associated with retrospective observational study designs is present. Second, the study period was relatively long to ensure a large study population, and changes in management and treatment practices during this period may have affected these results. Additionally, this study involved 5 international centers where reporting practices and differences in pathological evaluations may exist. However, this study has multiple strengths, including ensuring that T-stage was based on the size of the invasive component, not the overall cyst size, which is concordant with expert recommendation and only clearly defined in around 10% of published studies (15,30). Furthermore, the multi-institution study design also provides strength by making these findings more representative and generalizable.

This study serves as the largest evaluation and first validation of the T1 sub-staging recommendation for IPMN-derived PDAC. In this validation, we observed an association with advancing T1 sub-stage and progressively worsening histopathological features, overall survival, and recurrence, and only T2-4 tumors had an associated benefit from adjuvant chemotherapy. Sub-staging T1 IPMN-derived PDAC has significant prognostic value, and this study supports the incorporation of the proposed sub-staging system in IPMN-derived PDAC guidelines.

## Supplementary Material

djae166_Supplementary_Data

## Data Availability

The data underlying this article are international and multicentric in nature and require reasonable request and approved legal data transfer agreements with each participating center.
