# Supplementary material for: An international multi-institutional validation of T1 sub-staging of intraductal papillary mucinous neoplasm-derived pancreatic cancer
Source: J Natl Cancer Inst. 2024 Jul 19;116(11):1791–7. doi: 10.1093/jnci/djae166 (PMC11542988; doi:10.1093/jnci/djae166)

SUPPLEMENTARY MATERIAL

Supplementary Table 1: Univariable comparison between T1a and T1b for demographics and clinicopathologic information

Variable, n (%)	T1a (n=69)	T1b (n=50)	p-value
**Age>65**	44 (64)	32 (64)	0.979
**Female**	32 (46)	24 (48)	0.861
**Preoperative CA19-9**			
**Elevated**	12 (27)	18 (44)	**0.047**
**Normal**	32 (71)	22 (54)	
**Non-producer**	1 (2)	1 (2)	
**Unknown**	24	9	
**Operation**			
**Pancreatoduodenectomy**	36 (52)	30 (60)	0.694
**Distal pancreatectomy**	17 (25)	10 (20)	
**Total pancreatectomy**	16 (23)	10 (20)	
**R1-margin**	1 (1)	2 (4)	0.572
**Poor grade of differentiation**	11 (16)	7 (14)	0.805
**Tubular Subtype**	39 (62)	28 (62)	0.973
**Perineural Invasion**	6 (11)	9 (31)	**0.027**
**Positive Nodes**	2 (3)	8 (16)	**0.016**
**Adjuvant Chemotherapy**	22 (38)	21 (53)	0.153

Supplementary Table 2: Univariable comparison between T1a and T1c for demographics and clinicopathologic information

Variable, n (%)	T1a (n=69)	T1c (n=97)	p-value
**Age>65**	44 (64)	67 (69)	0.474
**Female**	32 (46)	50 (52)	0.511
**Preoperative CA19-9**			
**Elevated**	12 (27)	47 (53)	**0.006**
**Normal**	32 (71)	36 (41)	
**Non-producer**	1 (2)	5 (5)	
**Unknown**	24	9	
**Operation**			
**Pancreatoduodenectomy**	36 (52)	51 (53)	0.778
**Distal pancreatectomy**	17 (25)	20 (21)	
**Total pancreatectomy**	16 (23)	26 (27)	
**R1-margin**	1 (1)	20 (21)	**<0.001**
**Poor grade of differentiation**	11 (16)	21 (22)	0.325
**Tubular Subtype**	39 (62)	70 (76)	0.058
**Perineural Invasion**	6 (11)	23 (47)	**<0.001**
**Positive Nodes**	2 (3)	42 (43)	**<0.001**
**Adjuvant Chemotherapy**	22 (38)	50 (65)	**0.002**

Supplementary Table 3: Univariable comparison between T1b and T1c for demographics and clinicopathologic information

Variable, n (%)	T1b (n=50)	T1c (n=97)	p-value
**Age>65**	32 (64)	67 (69)	0.534
**Female**	24 (48)	50 (52)	0.684
**Preoperative CA19-9**			
**Elevated**	18 (44)	47 (53)	0.377
**Normal**	22 (54)	36 (41)	
**Non-producer**	1 (2)	5 (5)	
**Unknown**	9	9	
**Operation**			
**Pancreatoduodenectomy**	30 (60)	51 (53)	0.619
**Distal pancreatectomy**	10 (20)	20 (21)	
**Total pancreatectomy**	10 (20)	26 (27)	
**R1-margin**	2 (4)	20 (21)	**0.007**
**Poor grade of differentiation**	7 (14)	21 (22)	0.261
**Tubular Subtype**	28 (62)	70 (76)	0.091
**Perineural Invasion**	9 (31)	23 (47)	0.168
**Positive Nodes**	8 (16)	42 (43)	**0.001**
**Adjuvant Chemotherapy**	21 (53)	50 (65)	0.191

Supplementary Figure 1: Overall Survival by T-stage and Ductal Type. Kaplan-Meier survival curves for overall survival stratified by T-stage and ductal type. All ductal type comparisons within each T classification were not statistically significant (p>0.05).

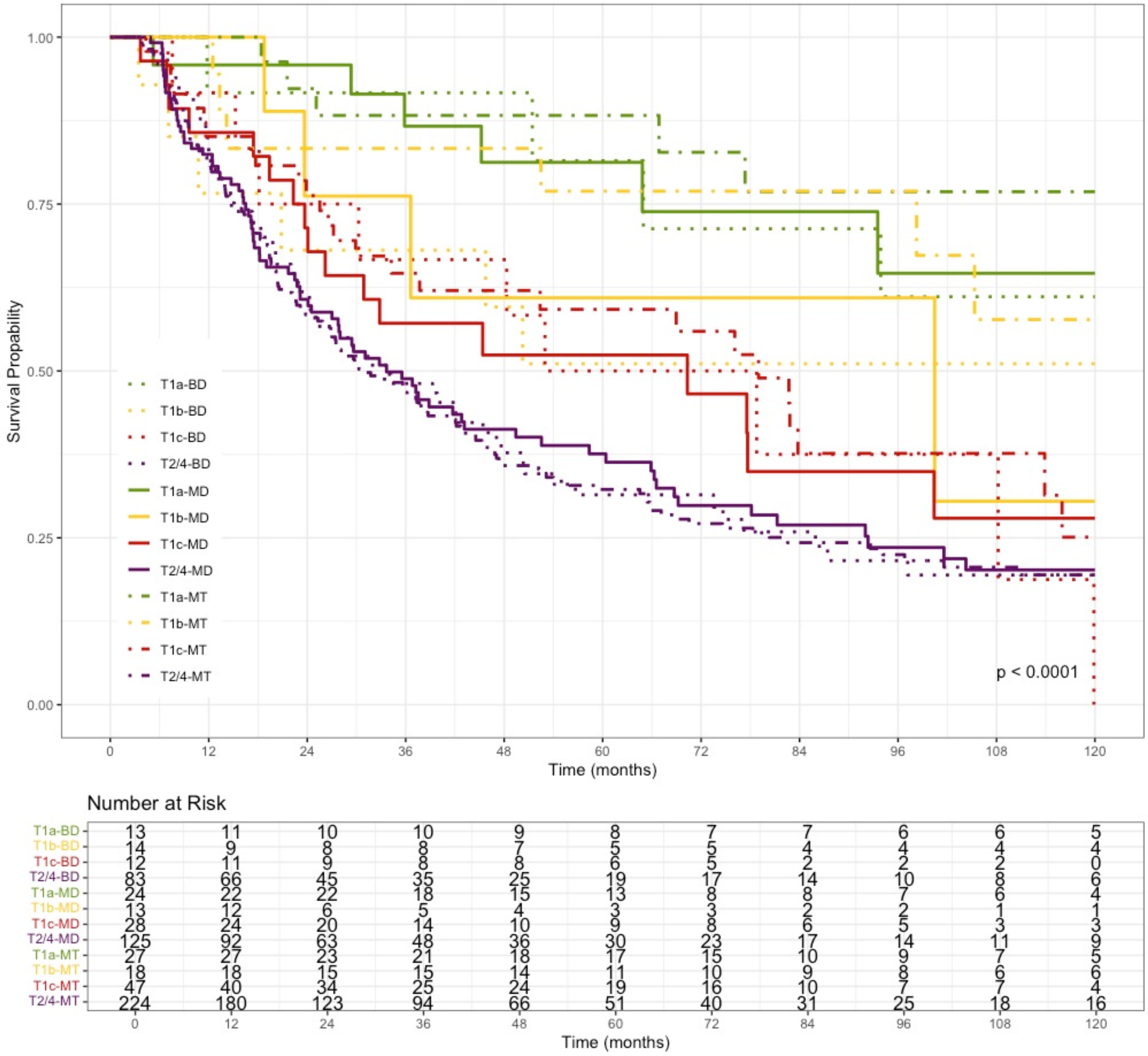

Supplementary Figure 2: Multivariable Cox-regression for Overall Survival. Forrest plot depicting hazard ratios and 95% confidence intervals of variables significant on Cox-regression analysis including tumor size (per centimeter).

Variable		Hazard ratio	P value
**Age**	≤65	Reference	
	>65	1.44 (1.11, 1.86)	0.006
**Tumor Size**		1.06 (1.03, 1.10)	<0.001
**pN-Stage**	pNneg	Reference	
	pNpos	2.17 (1.66, 2.83)	<0.001
**Resection Margin**	R0	Reference	
	R1	1.42 (1.09, 1.85)	0.010
**CA19-9**	normal	Reference	
	elevated	1.62 (1.20, 2.18)	0.002
	non-secreter	1.93 (1.06, 3.52)	0.033
	not available	1.95 (1.33, 2.84)	<0.001
**Adjuvant Chemotherapy**	no	Reference	
	yes	0.72 (0.55, 0.95)	0.021

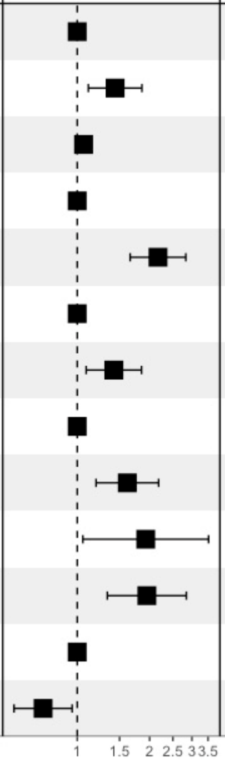

Supplementary Figure 3: Overall Survival by T-stage and Adjuvant Chemotherapy. Kaplan-Meier survival curves for overall survival stratified by T-stage and receipt of adjuvant chemotherapy with overall p-value.

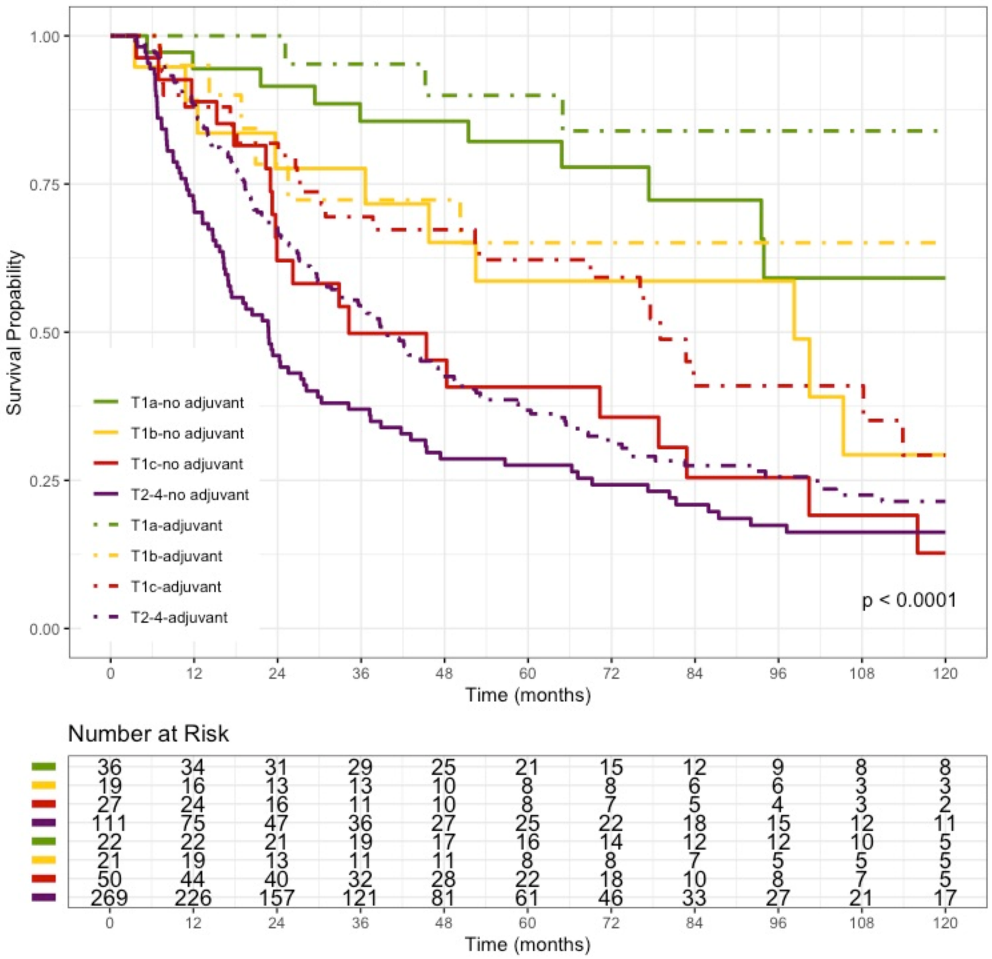

Supplement: djae166_Supplementary_Data [file djae166_supplementary_data.pdf]
